# Prevalence of stroke, associated risk factors and stroke related physical, mental, and economic burden in the Republic of Georgia

**DOI:** 10.1177/23969873221101987

**Published:** 2022-05-25

**Authors:** Tamar Akhvlediani, Nana Gelenidze, Tamar Janelidze, Tamar Gudadze, Irine Pkhakadze, Alastair Webb, Zaza Katsarava

**Affiliations:** 1 American MD Program, Faculty of Medicine, Tbilisi State Medical University, Tbilisi, Georgia; 2 Evex Medical Corporation, Tbilisi, Republic of Georgia; 3 Akaki Tsereteli State University, Kutaisi, Republic of Georgia; 4 Wolfson Centre for Prevention of Stroke and Dementia, University of Oxford, Oxford, UK; 5 Christian Hospital Unna, Unna, Germany; 6 Department of Neurology, University of Duisburg-Essen, Essen, Germany

**Keywords:** Stroke, prevalence, burden, eastern Europe

## Abstract

**Introduction:**

We investigated the prevalence, risk factors and physical, mental, and economic consequences of ischemic Janelidze and hemorrhagic stroke in the population of the Republic of Georgia.

**Materials and Methods:**

A population-based, cross-sectional study was conducted among 3036 adults residing in the Imereti Region of Georgia, selected using a multistage, probability proportionate-to-size, cluster sampling technique. Data were collected by medical students, using an interviewer-administered questionnaire. Diagnosis of stroke was confirmed by neurologists based on clinical examination and corroborated by documental evidence.

**Results:**

Of the targeted 3036 subjects, 2811 (92.6%) participated, of whom 1223 (43.5%) were women. Mean age of the sample was 49.7 (SD 15.2) years. The overall prevalence of stroke was 8.9%, the prevalence of ischemic stroke − 7.8% (95% CI 6.9–8.9) and of hemorrhagic stroke − 0.7% (95% CI 0.4–1.0). Ischemic stroke was more prevalent in males, while hemorrhagic stroke was more prevalent in females. Age, smoking, hypertension, and diabetes were associated with stroke. Stroke victims were young, many of them in the fifth decade of life. Sixty-five percent of them had a modified Rankin scale of three or greater, 25% were depressed, and 85% suffered cognitive impairment.

**Discussion:**

Stroke affected people and their families, experiencing a significant economic burden due to loss of the income and increase in out-of-pocket payment for post-stroke medical care.

**Conclusion:**

The stroke prevalence in the Republic of Georgia is higher than in Europe and is associated with a significant physical, mental, and economic burden.

## Introduction

Stroke remains the second leading cause of death and disability worldwide^1^ and among the three commonest causes of disability (disability-adjusted life years, DALYs) in the EU.^2^ Overall, approximately 70% of stroke deaths and about 80% of DALYs occur in low- and middle-income countries.^2^ Republic of Georgia is among such countries, located in the South Caucasus. The population of Georgia is 3.7 million, of which about 57% lives in urban setting. The population with the age of 65 or more represents 14.3% of the total population of the country.^3^ In 2014, in partnership with the Institute for Health Metrics and Evaluation, the National Center for Disease Control and Public Health of Georgia (NCDC) started the Global Burden of Disease study. In terms of the number of years of life lost (YLLs) due to premature death in Georgia, ischemic heart disease, cerebrovascular disease, and other cardiovascular and circulatory diseases were the highest-ranking causes in 2010.^4^ According to this study, years lost to disability (YLD) due to stroke scored the highest among 10 other causes, indicating that measures to prevent and treat this disease are insufficient and this disease requires mobilization and improvement of knowledge and resources in Georgia.

Despite this background, there still is a big gap in stroke surveillance in Georgia, and incidence and prevalence of this disease are unknown. There is only one study, conducted in 2004, which evaluated the stroke incidence in the Urban population of one suburb of the Capital city, Tbilisi. It reported the crude annual incidence rate of 89 (95% CI, 74–106) for ischemic stroke and 44 (95% CI, 34–57) for intracerebral hemorrhage, per 100 000 inhabitants.^5^ The study did not cover the population outside of the Capital of the Country and the prevalence rates remained unknown.

To reduce this gap, we conducted a population-based stroke prevalence study in Imereti, the largest region in Georgia. We aimed to investigate the point prevalence of stroke and vascular risk factors; evaluate the physical, mental, and socio-economic burden of stroke victims and their social environment; define the quality of medical service uptake in the population and assess the population’s awareness of stroke, ability of stroke recognition, and knowledge of emergency measures to be taken.

## Methods

Ethical approval for the study was obtained from the Ethics Committee of the Akaki Tsereteli State University, Kutaisi, Georgia. A written informed consent was obtained from all subjects. If the volunteer was not lucid, the consent was received from the legal guardian.

### Sampling plan

The population of Imereti region is 534,000. Among them 258,000 (48.4%) live in urban area and 275,000 (51.6%) – in rural area. The biggest city in this region is Kutaisi, with the population of 148,000. The sampling Frame was based on the 2014 General Population Census data for Georgia. Sampling from the target population was performed by multi-stage sampling approach.

The population of interest was the Imereti region of Georgia, consisting of 11 municipalities and the city of Kutaisi. The probability proportional to size approach was used for sampling. In urban setting, the streets were randomly selected. In each settlement, urban and rural, the starting point was selected randomly, and then every fifth household was approached. With estimated prevalence of 0.5% for hemorrhagic stroke, confidence coefficient of 95%, and the precision of 0.003, with 15% added for non-responders, the total sample size of 3000 individuals was calculated.

We included adults older than 18 years of both genders. Participants who refused to participate were not included in the study. All individuals of the household, meeting inclusion criteria, were offered to participate in the study. If any eligible household member was not at home at the time of the study team visit, the study team performed two more attempts, including visits in the evening and on week-ends, to meet them and give them an opportunity to participate.

### Screening for stroke victims and vascular risk factors

Screening of the population was performed by students of medical faculty of Akaki Tsereteli University in Kutaisi. Surveyors were trained by Dr. Janelidze and Dr. Akhvlediani prior to the study initiation. Screening was performed using pre-defined screening questionnaires which included questions about age, gender, socio-demographic characteristics such as education, employment, income, and surveyors’ overall impression of wealth, stroke related behavioral factors such as smoking, drinking, exercise, habits and diet, personal history of stroke or myocardial infarction, and chronic diseases such as hypertension, diabetes, dyslipidemia, atrial fibrillation, and intake of medication. For the stroke screening, we used the World Health Organization definition of stroke.^6^ Study participants were asked if they “ever experienced one or more of the following symptoms that was of sudden onset and lasted for more than 24 h such as (a) loss of strength (weakness) and/or numbness of face, arm and/or leg on one side, (b) difficulty speaking or understanding, (c) blurring of vision, double vision, or loss of vision in one eye or part of the visual field, (d) severe and unusual headache (worst ever), and (e) loss of balance. Subjects were also asked if they were diagnosed having stroke by a doctor. Those who responded “yes” to one the screening questions was considered as screening positive.

### Verification of cases

Screening positives (subjects suspected to have had stroke) were investigated face-to-face by neurologists from the EVEX Referral Hospital in Kutaisi. All subjects received complete neurological examination and all available medical documents were reviewed and if unavailable were actively searched and reviewed in the hospitals or clinics of general practitioners. The diagnosis of stroke and its subtypes (ischemic or hemorrhagic) were established based on self-reported history, neurological examination, and available medical documents including imaging reports.

Physical disability of stroke victims was assessed using the National Institutes of Health (NIHSS) stroke scale and modified Rankin scale (mRS). The Hospital Anxiety and Depression Scale (HADS) questionnaires^7^ were used to investigate psychological status of stroke victims and their spouses and the Mini-Mental-Scale^8^ was used to assess possible cognitive impairment. We also asked about financial burden due to stroke, for example, amount of money lost due to stroke and monthly out-of-pocket expenses for medication and medical services.

## Statistical analysis

We analyzed the following socio-demographic characteristics: age, gender, education (high = university or higher vs low), marital status (living alone vs living with partner), employment (employed, unemployed, student, pensioner), income in the previous year (low = less than 1000 Georgian Lari (GEL) = 300 USD per month vs high = more than 1000 GEL/300 USD per month), surveyors’ overall impression (wealthy, middle wealth, and poor). Vascular risk factors were defined as smoking (yes vs no), alcohol intake (regular = 1–2 times per week or more frequently vs no = once per week or less), salt intake (high = adding salt regularly vs low), healthy lifestyle (yes = walking or doing sports three or more times per week, no = else), family history of stroke (yes/no), myocardial infarction (yes/no), and peripheral artery disease (yes/no) and vascular diseases reported by study participants such as diabetes (yes/no), hypertension (yes/no), atrial fibrillation (yes/no), hypercholesterolemia (yes/no), and personal history of stroke (yes/no), myocardial infarction (yes/no), and peripheral artery disease (yes/no).

Data were analyzed using the SPSS software. Categorical variables are reported as numbers and percentages and continuous variables as means and standard deviations. Pearson’s chi-square test or Fisher’s exact test when appropriate were used to compare differences in categorical variables. Whereas, the Student *t*-test or Mann-Whitney U test, a non-parametric test, were used to compare differences for continuous variables. To investigate potential socio-demographic characteristics (e.g., gender, age) and epidemiological risk factors associated with stroke, unadjusted odds ratios (ORs) along with their 95% CIs were calculated using the logistic regression model.

## Results

The study was performed between July 2018 and completed by September 2021. It is worth to mention that the first thrombolysis in Georgia was performed in 2015.

A total of 2811 people out of 3036 targeted (predefined 3000) were recruited for the study, giving a response rate of 92.6%. Of them, 1223 (43.5%) were women. The mean age of participants was 49.7 (SD, 15.2) years with a median age of 50 years. The years of local residency ranged between 0 and 79 years. During their life span, according to their memories, 496 individuals have died due to stroke or its consequence.

**Figure 1.**
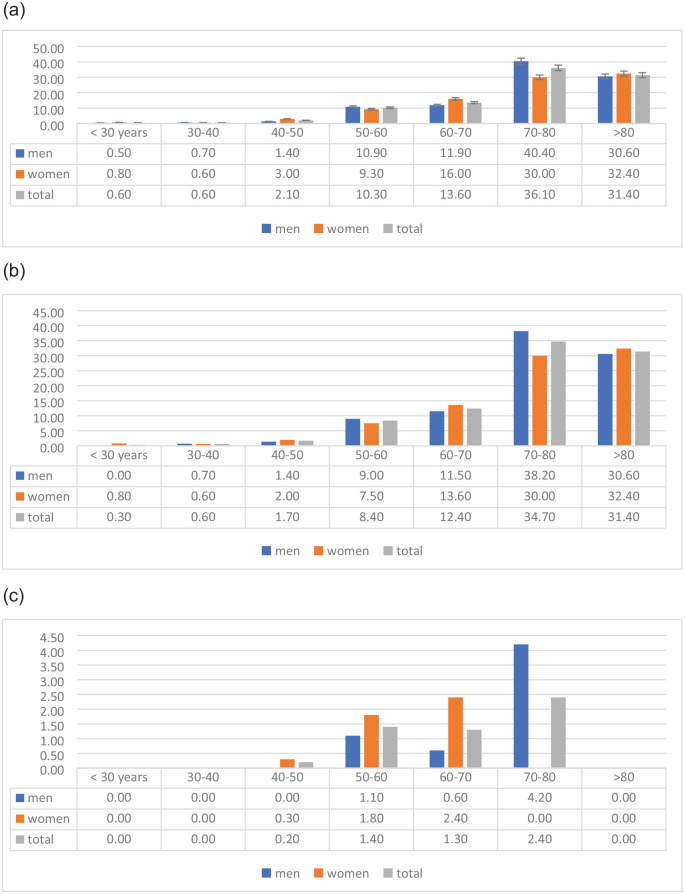
Prevalence of total (a), ischemic (b), and hemorrhagic (c) stroke stratified by gender and age, presented as percentages in different age groups.

The socio-demographic characteristics and vascular risk factors of the study sample is given in Figure 1.

According to students’ evaluation, 380 (13.5%) subjects were positive for stroke-screening. All these subjects were further examined by neurologists. Medical records were available in 193 cases, in 37 cases medical documents were collected retroactively and in 21 cases records were not available. Finally, 251 of total subjects (8.9%, 251 of 2811) were confirmed to have suffered stroke in the past. Ischemic stroke was confirmed by existing imaging report in 220 cases (131 males and 89 females). Of those, 183 cases occurred in the years 2000–2014 and 37 strokes occurred between 2015 and 2020. None of them received systemic rTPA or thrombectomy.

The crude life-time prevalence of ischemic stroke was 7.8% (95% CI 6.9–8.9) and the gender adjusted life-time prevalence was 7.3% (95% CI 5.6–8.9) in females and 8.3% (95% CI 7.0–9.7) in males. Ninety-six cases had the first stroke and 155 patients had recurrent stroke. Hemorrhagic stroke was confirmed by existing imaging report in 19 cases resulting in the crude life-time prevalence of hemorrhagic stroke of 0.7% (95% CI 0.4–1.0) and the gender adjusted life-time prevalence of 0.8% (95% CI 0.4–1.5) in females and 0.6% (95% CI 0.4–1.1) in males. In 12 cases, the type of stroke remained unknown. The prevalence of ischemic and hemorrhagic stroke stratified by gender and age is given in Figure 1(a) to (c).

Adjusted ORs for the individual risk factors by multivariable logistic regression model are shown in Table 1. The multivariable logistic regression showed that multiple characteristics were significantly associated with stroke, including age, smoking, hypertension, diabetes, and history of stroke. We did not calculate a regression model for hemorrhagic stroke because of a small number of cases. Notably, the prevalence of hemorrhagic stroke was higher in women than in men, peaking in the fifth and sixth decades. The prevalence of self-reported hypertension did not differ between the stroke and reference groups and approximately 75% of subjects affected by hypertension treated it irregularly, only when pressure was elevated.

**Table 1.** Socio-demographic features and vascular risk factors of the study sample.

	Overall stroke, N (%)	Ischemic stroke, *N* (%)	Hemorrhagic stroke, *N* (%)	Reference population, *N* (%)	Total, *N* (%)
Variables	251 (8.9)	220 (7.8)	19 (0.7)	2560 (91.1)	2811
**Age (mean; SD)**	**64.6 (12.1)**	**65.8 (11.7)**	**58.3 (9.6)**	**48.2 (14.7)**	**49.7 (15.2)**
**OR, 95% CI**	**1.08 (1.07–1.09)**	**1.09 (1.08–1.1)**			
Gender					
Men	148 (9.3)	131 (8.2)	9 (0.6)	1440 (90.7)	1588
Women	103 (8.4)	89 (7.3)	10 (0.8)	1120 (91.6)	1223
Education (*N*; %)					
High	119 (7.9)	106 (7.0)	7 (0.5)	1390 (92.1)	1509
Low	132 (10.1)	114 (8.8)	12 (0.9)	1170 (89.9)	1302
Partnership (*N*; %)					
Living alone	64 (7.4)	57 (6.6)	3 (0.3)	796 (92.6)	860
Living in partnership	187 (9.6)	163 (8.4)	16 (0.8)	1764 (90.4)	1951
Employment (*N*; %)					
Employed	75 (5.2)	60 (4.2)	10 (0.7)	1369 (94.8)	1444
Unemployed	60 (6.8)	49 (5.5)	6 (0.7)	824 (93.2)	884
Student	2 (2.2)	1 (1.1)	0	90 (97.8)	92
Pensioner	114 (29.2)	110 (28.1)	3 (0.8)	277 (70.8)	391
Income (*N*; %)					
High	147 (8.6)	126 (7.4)	12 (0.7)	1565 (91.4)	1712
Low	104 (9.5)	94 (8.6)	7 (0.6)	995 (90.5)	1099
Wealth (*N*; %)					
Wealthy	61 (7.8)	46 (5.9)	11 (1.4)	722 (92.2)	783
Middle wealth	147 (9.5)	133 (8.6)	8 (0.5)	1403 (90.5)	1550
Poor	43 (9.0)	41 (8.6)	0	435 (91)	478
**Smoking**					
**Yes**	**60 (11.9)**	**43 (8.5)**	**12 (2.4)**	**446 (88.1)**	**506**
**No**	**191 (8.3)**	**177 (7.7)**	**7 (0.3)**	**2114 (91.7)**	**2305**
**OR, 95% CI**	**1.6 (1.1–2.2)**				
Alcohol					
Regular	50 (9.4)	44 (8.3)	5 (0.9)	480 (90.6)	530
Irregular	201 (8.8)	176 (7.7)	14 (0.6)	2080 (91.2)	2281
Salt					
Low salt	62 (9.3)	52 (7.8)	4 (0.6)	606 (90.7)	668
High salt	189 (8.8)	168 (7.8)	15 (0.7)	1954 (91.2)	2143
Healthy lifestyle					
Healthy	82 (8.6)	70 (7.4)	5 (0.5)	870 (91.4)	952
Non healthy	169 (9.1)	150 (8.1)	14 (0.8)	1690 (90.9)	1859
Hypertension					
Yes	150 (14.5)	136 (13.1)	8 (0.8)	888 (85.5)	1038
No	101 (5.7)	84 (4.7)	11 (0.6)	1672 (94.3)	1773
OR, 95% CI	1.9 (1.4–2.5)	1.93 (1.4–2.7)			
Atrial fibrillation					
Yes	67 (8.4)	56 (7.0)	8 (1)	735 (91.6)	802
No	184 (9.2)	164 (8.2)	11 (0.5)	1825 (90.8)	2009
**Diabetes**					
**Yes**	**51 (21.4)**	**45 (18.9)**	**4 (1.7)**	**187 (78.6)**	**238**
**No**	**200 (7.8)**	**175 (6.8)**	**15 (0.6)**	**2373 (92.2)**	**2573**
**OR, 95% CI**	**1.9 (1.4–2.5)**	**1.8 (1.2–2.8)**			
Hypercholesterolemia					
Yes	33 (12.1)	33 (12.1)	0	239 (87.9)	272
No	218 (8.6)	187 (7.4)	19 (0.7)	2321 (91.4)	2539
**History of stroke**					
**Yes**	**50 (28.2)**	**44 (24.9)**	**4 (2.3)**	**127 (71.8)**	**177**
**No**	**201 (7.6)**	**176 (6.7)**	**15 (0.6)**	**2433 (92.4)**	**2634**
**OR**, **95% CI**	**3.7 (2.4–5.8)**	**3.8 (2.4–6.0)**			
History of MI					
Yes	22 (7.9)	18 (6.5)	2 (0.7)	255 (92.1)	277
No	229 (9.0)	202 (8.0)	17 (0.7)	2305 (91.0)	2534
History of peripheral vessel disease					
Yes	22 (7.9)	18 (6.5)	2 (0.7)	255 (92.1)	277
No	229 (9.0)	202 (8.0)	17 (0.7)	2305 (91.0)	2534

Numbers of overall strokes and of the reference population sum up to the total number. Numbers of ischemic and hemorrhagic strokes result in the numbers of overall stroke. OR and 95% CI are calculated comparing stroke samples to the reference group. ORs for hemorrhagic stroke were not calculated because of small number of cases. Only statistically significant associations are shown, marked in bold.

Stroke victims reported to have suffered during acute stroke attack mostly weakness, loss of balance, speech, or visual problems and, less frequently, headache. Eighty percent of stroke-affected subjects, or their family members called ambulance and patients were transferred to the hospital. The time span between the symptom onset and admission to the hospital remained unknown.

The demographic and clinical characteristics of ischemic and hemorrhagic stroke cases and its physical, emotional, and economic consequences are given in Table 2. Two hundred thirty-eight (97.5%) of stroke victims reported that their life was significantly affected by the stroke. At the time of survey, 164 (65.3%) individuals had a modified Rankin scale score of 3 or greater. Many of them were cognitively and emotionally impaired: 202 (80.5%) individuals suffered mild cognitive impairment, additional 13 (5.2%) subjects were demented, and 62 (24.7%) individuals were depressed. Stroke victims and their families reported to have been experiencing a significant economic burden because of stroke: the families lost an average 137 GEL (44USD) per month, while they have been paying about 73 GEL (24USD) per month out-of-their pocket for medication and medical services.

**Table 2.** Physical, mental, emotional, and economic consequences of stroke for affected individuals and their families.

Characteristics	Stroke		
	Total (251)	Men (148)	Women (103)
Age (mean; SD)	64.6 (12.1)	65.22 (11.7)	63.71 (12.5)
Stroke consequences			
NIHSS (mean; SD)	4.14 (1.9)	4.26 (2.0)	3.95 (1.8)
mRS (median, max–min)	2.26 (5–0)	2.33 (5–0)	2.17 (5 - 0)
mRS ⩾ 3, *N* (%)	164 (65.3)	101 (68.2)	63 (61.2)
HADS D (mean, SD)	7.83 (1.6)	7.8 (1.6)	7.87 (1.5)
HADS A (mean, SD)	4.44 (1.7)	4.47 (1.7)	4.41 (1.7)
Depression, (*N*, %)			
Yes, HADS-D ⩾ 8	62 (24.7)	34 (23)	28 (27.2)
No	189 (75.3)	114 (77)	75 (72.8)
Anxiety, (*N*, %)			
Yes, HADS-A ⩾ 8	3 (1.2)	2 (1.4)	1 (1)
No	248 (98.8)	146 (98.6)	102 (99)
MMSE, dementia (*N*, %)			
Yes (MMSE < 23)	13 (5.2)	6 (4.1)	7 (6.8)
Mild cognitive impairment (MMSE 23–27)	202 (80.5)	123 (83.1)	79 (76.7)
No	36 (14.3)	19 (12.8)	17 (16.5)
Stroke consequences of the social environment			
Spouse HADS D (mean, SD)	4.25 (1.7)		
Spouse HADS A (mean, SD)	4.1 (2.0)		
Spouse depression (*N*, %)			
Yes, HADS-D ⩾ 8	5 (2)		
No	246 (98)		
Spouse anxiety (*N*, %)			
Yes, HADS-A ⩾ 8	6 (2.4)		
No	245 (97.6)		
Income lost (mean, SD)	136.37 (123)	130.41(113.34)	144.95 (135.83)
Money spent for the treatment per month (mean, SD)	72.27 (24.7)	72.3 (26)	72.23 (22.79)

NIHSS: National Institute of Health Stroke Scale; mRS: modified Rankin Scale; HADS: Hospital Anxiety and Depression Scale; MMSE: Mini Mental Status Exam.

## Discussion

In this population-based study we assessed the prevalence of stroke and related physical mental and economic burden in the Republic of Georgia. The prevalence of overall stroke was 8.9% of ischemic stroke – 7.8% and of hemorrhagic stroke − 0.7%. We revealed a severe physical and mental disability of stroke victims and a significant economic burden of their families.

The prevalence of stroke in Europe is 1.8%.^9^ The studies from the UK and the US reported a range from 1.7% to 2.6%,^10^ a German study provided the prevalence of 2.9%.^11^ Similar numbers were reported in Asian Countries, for example, 1.4% in Indonesia,^12^ 3.1% in China,^13^ and 2.2% in the neighbor Turkey.^14^ No information is available about the prevalence of ischemic stroke in other neighbor countries such as Armenia, Azerbaijan, and Russia. Our study revealed the point prevalence of ischemic stroke of 7.8%, which is three times higher than in comparable studies in Europe, US, and Asia as well as in the neighboring Turkey.

In our study, age was identified as an important risk factor for stroke, like elsewhere.^15^ In contrast to Europe, stroke victims in Georgia were younger, over 10% of cases occurring between the ages of 50 and 60.^11^ Vascular risk factors, such as hypertension, diabetes, smoking, and history of stroke were identified to be associated with ischemic stroke. The prevalence of vascular risk factors such as dietary risk factors, smoking, alcohol use, high blood pressure is higher in Georgia than in the EU, as reported previously.^16^ Our study confirmed these findings, which explains the high prevalence of ischemic stroke, especially in the young.

Overall, the prevalence of intracerebral hemorrhage is about 0.1–0.2%.^2^ In our study, we revealed a significant higher number of 0.7%. The prevalence was especially high in 50–60-year-old men and women. The distribution of prevalence rates in our population peaks in the fifth and sixth decades both in men and women, which is unusual as men tend to be more frequently affected than women.^17^ The Georgian rates are higher than 0.24%–0.3%, detected in the neighboring Turkey.^14,18^ High blood pressure is the most important modifiable risk factor for all types of stroke. The prevalence of hypertension is high in Georgia and contributes to the high prevalence of the intracerebral bleedings. In addition, hypertension is not adequately treated in Georgia,^19^ since about 75% of respondents treated it irregularly, only if elevated, therefore increasing the risk of cerebral bleeding, again also at a relatively young age.

Stroke resulted in a significant physical, emotional, and economic burden of its victims and their families. About 60% of stroke survivors were moderately or severely disabled, about 25% of them were depressed and about 85% cognitively impaired. On average, families lost an income of about 42 USD per month and have been spending approximately 21 USD per month for the treatment. A population based south London stroke registry reported that 21% of stroke victims survived 15 years after the first ever stroke. Of them, 14% had moderate disability and 15% were severely disabled. The prevalence of cognitive impairment was 30.0%, depression 39.1%, and anxiety 34.9%, and survivors reported greater loss of physical than mental quality of life.^20^ Among the 10-year stroke survivors in Sweden, the need of assistance with mobility and self-care was reported by 14% and with usual activities by 22%. Moderate anxiety/depression was reported by 28% and high degree only by 1%.^21^ Specifically, the poststroke cognitive impairment ranged between 16% and 22% in different studies depending on methodology^22,23^ and the prevalence of poststroke depression was estimated about 25%.^24,25^ The physical outcome in our study, measured as the modified Rankin Scale score of 3 or greater, is worse than reported. This finding is not surprising since the modern stroke treatment facilities such as stroke units using systemic thrombolysis and thrombectomy have only been established in the Republic of Georgia very recently and do not cover the entire Country. For example, in the entire region of Imereti only one hospital is equipped with a state-of-the-art stroke unit, certified by the European Stroke Organization and provides systemic thrombolysis and thrombectomy. A countrywide network including pre-hospital ambulance system and regional stroke units referring selected patients to the neurovascular centers are not established yet. In addition, public awareness of stroke warning signs and appropriate actions to be taken (e.g. calling ambulance) is low, as was the knowledge and readiness of the prehospital ambulance doctors and nurses, which results in a significant delay at the prehospital level, which resulted in a very low rate of systemic thrombolysis and thrombectomies.^26^ The prevalence of cognitive impairment in our population, measured using Mini-Mental-Scale, which is known to be insensitive compared to more specific instruments^27,28^ was much higher than reported, very likely due to untreated hypertension.

Stroke was associated with a significant economic burden on an individual as well as a household and a community level. Even in affluent societies, higher pre-stroke income cannot protect from hardships after stroke.^29^ Our study shows that, on average, the household monthly income loss was 137 GEL (44 USD), which represents 61% of the subsistence minimum in Georgia for an adult male (estimated as 223 GEL (75USD) in December 2021, according to the statistics department of Georgia).^30^ A reported out-of-pocket payment of 73 GEL (24 USD) for post-stroke medications and medical services further increases the overall income loss. If we take into account that the household income in 36% of the study population was less than 1000 GEL, it is clear that households are risking to experience catastrophic health expenditures.

The aim of this study was to estimate the current point prevalence of stroke and evaluate stroke-associated risk factors. This has not been done in a population-based study in the Republic of Georgia before. Our study sample was large enough and population-based, achieved the response rate of 93% and therefore the results are generalizable. The two-step approach with face-to-face screening by medical students and subsequent neurological examination by neurologists ensured the high quality of data collection. The results are revealing high prevalence of stroke, especially in the young, and a significant burden as a result to it. The study also unveiled the consequences of a poor quality of primary care and the lack of adequate stroke care in the Country.

The limitations of the study are that we studied the population of western Georgia only. The mean age, women-to-men ratio, cultural and habitual attitudes, and quality of medical care in this region is more or less comparable with other parts of the Country, the socio-economic status might be higher, and the quality of medical care might be somewhat but not substantially better in Tbilisi, the Capital of Georgia. Therefore, caution is needed when extrapolating this data to the entire country. We defined stroke cases mainly based on CT (not MRI) performed during the hospital stay and therefore we might have underestimated minor strokes. In this case the prevalence rates of stroke would be even higher than reported. In addition, we cannot report reliably how many stroke victims died of stroke and its consequences in the population of interest. Therefore, the clinical outcome of stroke is very likely biased and underestimates the true prevalence. We did not examine the vascular risk factors, for example, we did not measure blood pressure, cholesterol, and sugar in blood, did not record ECG and simply relied on information provided by the study subjects. We, therefore, very likely did not reliably assess diabetes or atrial fibrillation. Furthermore, because of the cross-sectional design of the study we evaluated associations between potential vascular risk factors and stroke and their possible consequences such as depression and dementia but not causal relationships as it would only be possible in a longitudinal cohort investigation.

Stroke has been neglected for many years in Georgia. During the last 5 years several stroke centers have been established which provide systemic thrombolysis and thrombectomy.^26^ However, a countrywide network including pre-hospital ambulance system and regional stroke units referring selected patients to the neurovascular centers are not established yet. To estimate costs of potential services, it is necessary to have information on the stroke prevalence and incidence. The knowledge of the physical, mental, and economic burden by stroke would complete the picture. We think the current manuscript provides an additional piece of information to the whole picture of stroke burden in the Republic of Georgia and contributes to a better understanding of stroke burden in Eastern Europe.
